# Air pollution, medical resources and cardiometabolic diseases: an empirical analysis from health inequality prospective

**DOI:** 10.1186/s12889-026-26829-z

**Published:** 2026-03-09

**Authors:** Wenli Liu, Junjie Feng, Fang Xie, Kunpeng Liu

**Affiliations:** 1 Shaoxing Maternity and Child Health Care Hospital, Shaoxing, 312000 China; 2 Maternity and Child Health Care Affiliated Hospital, Shaoxing University, Shaoxing, 312000 China; 3 School of Public Affairs, Zhejiang University, Hangzhou, 310058 China

**Keywords:** Air pollution, Cardiometabolic diseases, Urban–rural inequality, Practicing physicians

## Abstract

Air pollution has been linked to an increased risk of cardiometabolic diseases (CMDs) among middle-aged and elderly individuals. However, few large-scale empirical studies have tested this correlations base on Chinese samples, and research on the urban–rural differences remains limited. This study aims to explore the relationship between air pollution (specifically PM2.5) and the risk of CMDs in middle-aged and elderly individuals, examining urban–rural inequality and assessing the moderating effect of healthcare resources. Data from the fourth wave of the China Health and Retirement Longitudinal Study (CHARLS), combined with a high-resolution PM2.5 dataset and data on practicing physicians from local Chinese governments, were used. A logistic regression model was employed to analyze disease-specific risks, assess urban–rural heterogeneity, and test moderating effects. Robustness analysis was performed to enhance the reliability of the findings. The results showed that PM2.5 exposure is significant correlated with five CMDs: hypertension, diabetes, dyslipidemia, heart disease, and stroke. The impact posed by PM2.5 differed between urban and rural areas that rural residents showed more vulnerable to air pollution. Increasing practicing physicians was found to mitigate the impact of PM2.5 on CMDs in the targeted people, especially for rural residents. The study suggests that local government should pay more attention towards the health of rural residents rather than urban residents, and implementing differentiated prevention and control measures. The allocation of medical resources should be continuously optimized, with a focus on increasing practicing physicians, particularly in rural areas, to strengthen health protection capabilities. Developing a comprehensive urban–rural integrated medical service system will be crucial in elevating the standard of medical services in rural regions.

## Introduction

Air pollution, particularly fine particulate matter (PM2.5), is a leading global environmental risk factor for cardiometabolic diseases (CMDs), contributing to significant mortality and morbidity, [18, 26]. While the general pathophysiological link between PM2.5 and CMDs is well-documented, the health impacts are not uniformly distributed across different countries and regions. These impacts are critically mediated by socioeconomic factors, which shape both exposure to pollution and the capacity to mitigate its health consequences [1, 21].

In China, a primary axis of socioeconomic inequality is the urban–rural divide. This divide encompasses disparities in wealth, infrastructure, and access to resources, including healthcare. Consequently, the burden of air pollution on CMDs is likely distributed unevenly across urban and rural populations. However, existing research on CMDs and air pollution has predominantly focused on urban settings or specific diseases [18, 20] with comparative studies on urban–rural disparities among the middle-aged and elderly remain scarce.

Within this background, healthcare accessibility—particularly the availability of (local) medical service—emerges as a key modifiable factor that may alter the relationship between environmental exposure and health outcomes. Physicians are central to the prevention, management, and mitigation of CMDs [23]. Yet, it is unclear whether and how regional physician supply moderates the effect of PM2.5 on CMDs incidence, and whether this moderating role differs between urban and rural settings.

To address these interconnected gaps, this study investigates the association between long-term PM2.5 exposure and CMDs among middle-aged and elderly Chinese, explicitly testing for urban–rural differences. Furthermore, it examines whether the availability of practicing physicians mitigates the adverse health impacts of air pollution, and if this protective effect varies across the urban–rural areas.

## Data and methods

### Individual level data

**Fig. 1:**
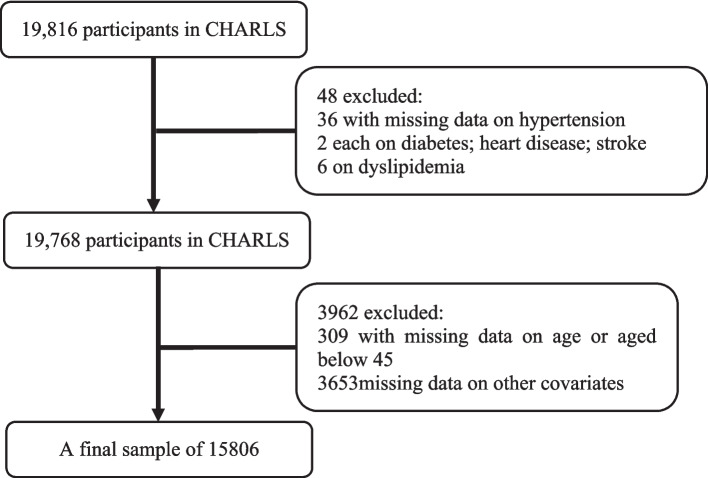
Flowchart of participant selection process

The China Health and Retirement Longitudinal Study (CHARLS) was conducted by Peking University, aiming to collect a set of high-quality micro-data representing households and individuals of Chinese individuals aged 45 and above, which was initiated its baseline survey in 2011, followed by subsequent surveys conducted in 2013, 2015, and 2018. These surveys encompassed a comprehensive coverage of 28 provinces, including autonomous regions and municipalities, across 150 counties and 450 communities (villages) nationwide. By the completion of the national follow-up in 2018, the sample included 19,000 respondents from 12,400 households [28]. Data for this study were extracted from the fourth wave of CHARLS, as air pollution and practicing physicians were most accurately recorded in 2018. A final sample of 15,806 individuals was retained for analysis, after the exclusion of participants with missing data on PM2.5, practicing physicians, health conditions, age, or covariates, as well as those under 45 years of age. The selection process of observations is displayed at Fig. 1.

### PM2.5 data

**Fig. 2:**
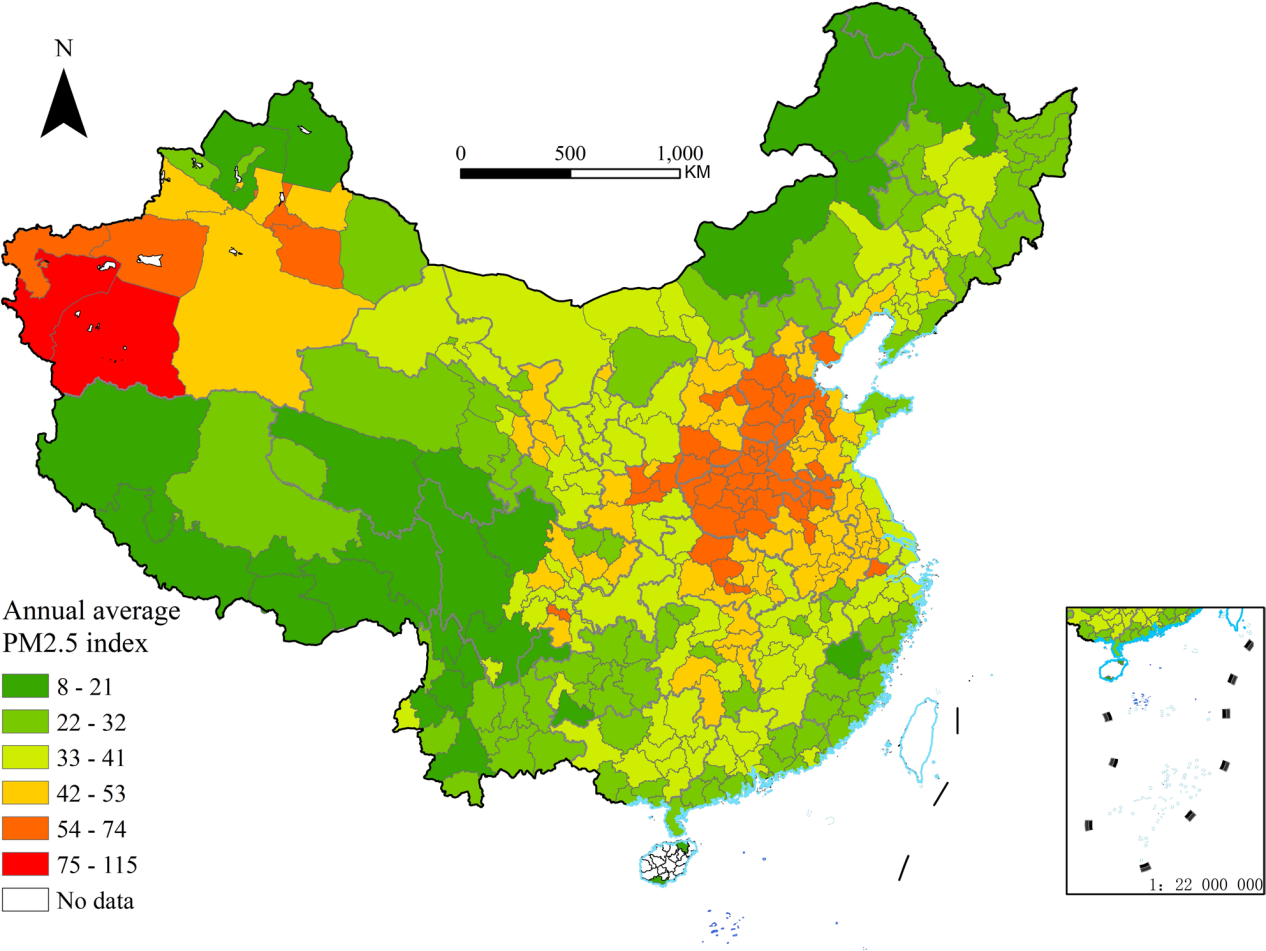
Annual average PM2.5 index in 2018

Considering that PM2.5 is an important indicator for measuring the degree of air pollution, and it is closely related to human health, we use the annual average ambient PM2.5 concentration at the city level as a proxy indicator for long-term population exposure. China High Air Pollutants (CHAP), which was released by Wei et al. [25] on the National Qinghai-Tibet Plateau Science Data Center website, employs artificial intelligence technology to generate seamless, nationwide PM2.5 data at a 1-km resolution based on extensive big data, ensuring reliability and authenticity. For this study, the CHAP dataset was supplemented with information from China's air quality monitoring stations. PM2.5 grid data and shapefiles sourced from the National Geographic Information Public Service Platform were employed, with the city level designated as the smallest geographical unit. Using ArcGIS software's zoning statistics tool, the annual average PM2.5 concentration was calculated for 342 administrative units (prefecture-level cities/regions/autonomous prefectures) in China for the year 2018 (see Fig. 2 below).

### AQI data

The Air Quality Index (AQI), which can be seen as another proxy variable for air pollution, serves as a comprehensive evaluation metric based on the concentrations of six pollutants—PM2.5, PM10, SO₂, NO₂, O₃, and CO—reflecting the severity of air pollution. This study relied on real-time monitoring data obtained from 1,605 air quality monitoring stations nationwide, which was collected and organized by Liu [11], effectively covering all prefecture-level cities. The annual average AQI for each city in 2018 was calculated from the daily average AQI and subsequently aggregated across 342 administrative units.

### Availability of (local) medical service

**Fig. 3:**
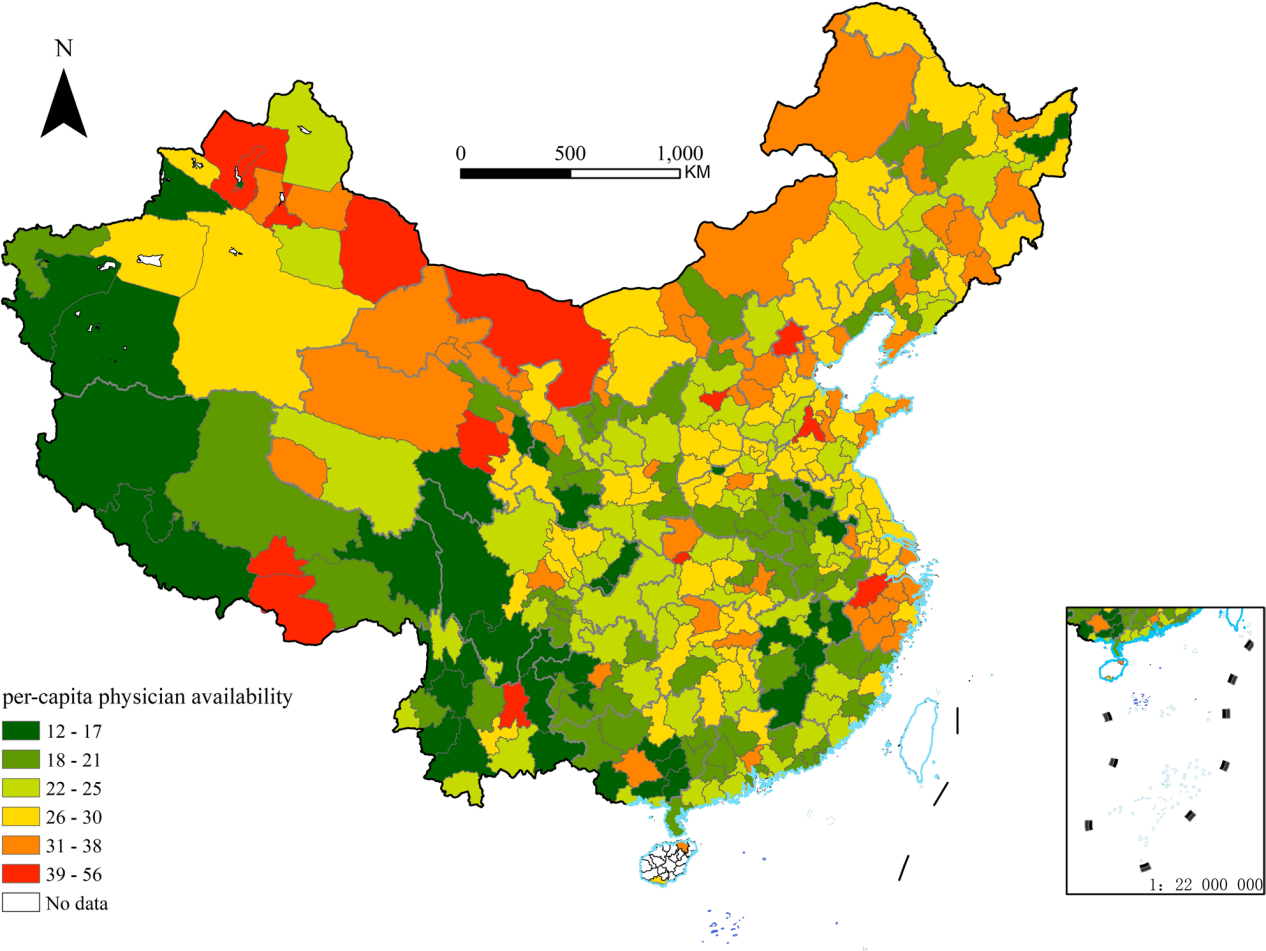
Per-capita physician availability in 2018

The accessibility of local public health services may be a key variable influencing the detection and treatment of chronic diseases among middle-aged and elderly populations exposed to air pollution, and the density of licensed physicians in a city serves as a reliable measure of local public health service accessibility. The *Statistical Communique on National Economic and Social Development* of China's prefecture-level cities is an authoritative source of local data, which provides annual data on the number of licensed physicians in the section of "Culture, Tourism, Health and Sports". To control for the impact of each city’s population size, we normalized the annual number of licensed physicians in each city by dividing it by the population size, and ultimately obtained the number of licensed physicians per 10,000 people in 342 prefecture-level cities in 2018, which is used as a proxy variable for measuring the accessibility of local public health services. This information was compiled into a dataset using ArcGIS software (see Fig. 3 below).

### Variable selection

#### Dependent variable

The “Health Status and Function” module in the CHARLS questionnaire includes 14 chronic diseases diagnosed by doctors. Respondents are asked whether they have received a diagnosis for the following conditions: hypertension; abnormal blood lipids; diabetes; cancer or malignant tumors; chronic lung disease; liver disease; heart problems; stroke; kidney disease; asthma; digestive system diseases; emotional, neurological, or mental issues; memory-related diseases; arthritis; or rheumatism. For this analysis, five CMDs were selected as dependent variables: hypertension, diabetes, dyslipidemia, heart disease, and stroke, which "0" indicate “no” while "1" indicate “yes”.

#### Independent variable

Based on the aforementioned discussion on air pollution data, PM2.5 was chosen as the primary air pollution indicator [9]. The annual average concentration of PM2.5 in various prefecture-level cities in China for 2018 was selected as the independent variable.

#### Control variables

These include individual-level variables such as gender ("1" indicates male; "2" indicates female), age, and marital status ("0" indicates unmarried or widowed; "1" indicates married), as well as socioeconomic status indicators like education level ("0" indicates unschooled; "1" indicates elementary school; "2" indicates middle school and above), social security.

("0" indicates “no” "1" indicates “yes”) and household consumption (logarithmically processed). At the regional level, the primary indicator employed is the annual gross domestic product (GDP) of each city (also logarithmically processed). To mitigate the skewness and approximate a normal distribution in regression, we applied a natural logarithm transformation to the data of household consumption and GDP. We use GDP instead of Per capita GDP, because GDP serves as a proxy for a city's overall economic activity and resource base, directly influencing the scale and capacity of public service investments—including funding for environmental governance and healthcare systems.

#### Moderating variable

Practicing physicians serves as a significant indicator of healthcare provision. Consequently, per-capita physician availability in 2018 was selected as the moderating variable.

### Empirical research design

In order to examine whether the risk of CMDs among middle-aged and elderly individuals systematically varied due to PM2.5 exposure, we use a logistic regression model to test the relationship. A reduced model was first constructed without control variables, after which an adjusted model was developed to include control variables such as gender, age, marital status, education level, household expenditure, annual city GDP, and other relevant factors. Robustness tests were subsequently conducted, substituting the independent variable from PM2.5 to the AQI and changing the regression model from logistic to probit regression.

The logistic model employed in this study is expressed as follows:

1 $$\mathrm{Cardiometabolic}\left(\mathrm{P}\left(\mathrm{Y}=1\right)\right)={\beta }_{0}+{\beta }_{1}\mathrm{PM}2.5+{{\delta}}{\mathrm{X}}$$

Cardiometabolic conditions encompass CMDs affecting middle-aged and elderly individuals. In this context, $P$ denotes the probability of disease development, ${\beta }_{0}$ represents the intercept term, and $PM2.5$ refers to the annual average PM2.5 index for prefecture-level cities in China in 2018. The coefficient ${\beta }_{1}$ reflects the magnitude of influence, while $\mathrm{X}$ incorporates additional influencing factors beyond PM2.5, including gender, age, marital status, education, family consumption, social security, and city GDP.

To further examining the potential health inequality between urban and rural residents, we exploit model (1) only in urban and rural samples respectively. For each equation, the coefficient ${\beta }_{1}$ became the magnitude of influence in the subgroup. By comparing the results of the two group, we can investigate the heterogeneity of the effects of PM2.5 exposure in urban and rural settings.

More importantly, as it was discussed before, the availability of sufficient medical resources is an important factor in effectively preventing and treating CMDs, and medical resources are mainly reflected in the number of local practicing physicians. Considering that Chinese residents generally seek medical treatment and seek medical attention within the city, cities are the basic units for Chinese residents to seek medical treatment and seek medical attention. Therefore, practicing physicians per 10,000 individuals of the city was integrated as a moderating variable, and logistic regression analyses were conducted to assess the moderating effects across the five categories of CMDs: hypertension, diabetes, dyslipidemia, heart disease, and stroke.

The logistic model considering the practicing physicians’ moderating effect is expressed as follows:

2 $$\begin{aligned}&\mathrm{Cardiometabolic}\left(\mathrm{P}\left(\mathrm{Y}=1\right)\right)={\beta }_{0}+{\beta }_{1}\mathrm{PM}2.5\\&+{\beta }_{2}\mathrm{PPs}+{\beta }_{3}\mathrm{PM2.5}\times \mathrm{PPs}+\delta \mathrm{X}\end{aligned}$$

In Eq. (2), $PPs$ represents practicing physicians per 10,000 individuals of the city where the interviewee is located. The coefficient ${\beta }_{2}$ reflects the magnitude of influence to the risk of CMDs while the local practicing physicians per 10,000 individuals change one percent. To be emphasized, the coefficient ${\beta }_{3}$ reflects the moderating effect through the air pollution exposure to the risk of CMDs process. If ${\beta }_{3}$ is negative, it is believed that the increase in the number of practicing doctors has reduced the risk of illness among middle-aged and elderly residents in the area due to air pollution. The meanings of other parameters are consistent with Eq. (1).

## Research results

### Descriptive statistics of main variables

The definitions of variables and descriptive statistical results are presented in Table 1. The mean and standard deviation of hypertension, diabetes, dyslipidemia, heart disease, and stroke are within a reasonable range. The average value of PM2.5 is 41.716, with a standard deviation of 14.170, indicating that there is a certain degree of air pollution in China, and there are significant differences between cities. The city with the smallest PM2.5 annual average index is only 15.336, while the largest is 73.780. Comparing the data in this article with existing literature [27, 29], the descriptive statistical results of other variables are within a reasonable range.

**Table 1:** Descriptive Statistics. Summary statistics of the study population, *N* = 15806

Variable	Definition	Mean	Std. dev	Min	Max
Hypertension	"0" indicates “no” "1" indicates “yes”	0.383	0.486	0	1
Diabetes		0.131	0.338	0	1
Dyslipidemia		0.224	0.417	0	1
Heart Disease		0.198	0.398	0	1
Stroke		0.071	0.258	0	1
PM2.5		41.716	14.170	15.336	73.780
Age		61.414	10.015	45	118
Gender	"1" indicates male; "2" indicates female	1.517	0.500	1	2
Marital Status	"0" indicates unmarried or widowed; "1" indicates married	0.865	0.342	0	1
Education	"0" indicates unschooled; "1" indicates elementary school; "2" indicates middle school and above	0.927	0.834	0	2
Social Security	"0" indicates “no” "1" indicates “yes”	0.966	0.180	0	1
Household Consumption	Logarithm of household consumption	10.260	1.003	3.178	15.394
GDP	Logarithm of the city GDP where the interviewee resides	16.977	0.918	15.070	19.605

### Baseline regression

To examine the impact of air pollution exposure on the risk of local residents’ CMDs, we matched the fourth wave of CHARLS data with the annual average PM2.5 concentration dataset of 342 administrative units (prefecture-level cities, regions, and autonomous prefectures) in China for 2018. Logistic regression analyses were conducted to examine the impact of PM2.5 on CMDs through Eq. (1) as baseline regression results.

A comparative analysis of the results (see Table 2) revealed that, the PM2.5 coefficients for all five cardiometabolic diseases (CMDs)—hypertension, diabetes, dyslipidemia, heart disease, and stroke—are positive and significant at the 1% level. This confirms that the positive association between PM2.5 exposure and CMD risk is statistically robust. A one-unit increase in PM2.5 concentration raises the log odds of hypertension by 0.007 (OR = exp(0.007)≈1.007), meaning a 0.7% increase in the odds of developing hypertension. This represents the weakest association among the five CMDs. In the meantime, each unit increase in PM2.5 elevates the log odds of dyslipidemia by 0.017 (OR≈1.017), equivalent to a 1.7% increase in disease odds, indicating that dyslipidemia has the strongest association among the five CMDs.

**Table 2:** Baseline regression table

**Variable**	Hypertension	Diabetes	Dyslipidemia	Heart disease	Stroke
PM2.5	0.007*** (0.001)	0.012*** (0.002)	0.017*** (0.001)	0.010*** (0.001)	0.010*** (0.002)
Age	0.049*** (0.002)	0.026*** (0.003)	0.023*** (0.002)	0.050*** (0.002)	0.056*** (0.003)
Gender (ref: Man)					
Woman	0.041 (0.035)	0.273*** (0.050)	0.240*** (0.041)	0.599*** (0.044)	−0.065 (0.067)
Marital status (ref: no)					
Yes	0.021 (0.052)	0.155* (0.074)	0.099 (0.062)	0.162** (0.062)	−0.060 (0.088)
Education (ref: unschooled)					
Elementary school	−0.008 (0.044)	0.015 (0.063)	0.155** (0.052)	0.230*** (0.054)	0.113 (0.082)
Middle school and above	0.062 (0.044)	0.210** (0.061)	0.525*** (0.050)	0.483*** (0.053)	0.232** (0.082)
Social security (ref: no)					
Yes	0.198* (0.096)	0.441** (0.156)	0.456*** (0.126)	0.264* (0.123)	0.458* (0.204)
Household consumption	0.043* (0.018)	0.083** (0.025)	0.180*** (0.021)	0.085*** (0.022)	0.096** (0.032)
GDP	−0.036# (0.019)	0.056* (0.027)	0.026 (0.022)	−0.255*** (0.024)	−0.240*** (0.038)
Constant	−3.846*** (0.387)	−6.599*** (0.555)	−6.522*** (0.457)	−2.407*** (0.480)	−3.940*** (0.740)
Observation	15805	15806	15805	15805	15806

^#^indicates *p* < 0.1. In parentheses are standard errors. The tables below are the same

*represents *p* < 0.05

**represents *p* < 0.01

***represents *p* < 0.001

Among the control variables, the coefficients of Age are positive and highly significant across all CMDs. A one-year increase in age raises the log odds of hypertension by 0.049 (OR≈1.050), meaning a 5.0% higher odds of hypertension—consistent with the biological mechanism that aging exacerbates cardiometabolic vulnerability.

Notably, the Gender control variables displays that female respondents show significantly higher odds of diabetes (β = 0.273, OR≈1.314), dyslipidemia (β = 0.240, OR≈1.271), and heart disease (β = 0.599, OR≈1.819) compared to males, with no significant difference in hypertension or stroke.

Household consumption is Positive and significant to getting five CMDS, suggesting that higher household consumption (a proxy for socioeconomic status) is associated with increased CMD risk, potentially reflecting lifestyle factors (e.g., high-calorie diets, sedentary behavior) linked to economic affluence.

Coefficients of city GDP vary by disease—negative for hypertension, heart disease***, and stroke***, indicating that higher urban economic development may mitigate these CMD risks possibly due to better environmental governance or healthcare access; it is positive for diabetes, which may relate to urban lifestyles tied to economic growth.

In terms of social security, having social security is associated with higher odds of all CMDs. The results are most likely driven by selection bias rather than a causal effect of social security itself, for individuals with higher health risks are more likely to enroll in social security.

### Robustness tests

Robustness tests were conducted by changing the main variables and model specifications. The first approach is to replace the main variable, and the 2018 annual AQI data for Chinese cities was utilized as the dependent variable. Although the AQI index incorporates PM2.5, it also encompasses multiple pollutants including PM10, SO₂, NO₂, O₃, and CO, providing a more systematic perspective on pollution exposure. Therefore, it remains effective for assessing the robustness of PM2.5 estimation results. As shown in Table 3, following this substitution, the estimated coefficients remained statistically significant at the 1% level. To maintain a consistent interpretation, the same percentage change transformation was applied to the alternative coefficients. When AQI is used as the alternative pollution measure in Table 3, A one-unit increase in PM2.5 concentration raises the log odds of hypertension by 0.006(OR = exp(0.006)≈1.006), meaning a 0.6% increase in the odds of developing hypertension. The similarity in effect magnitude between the PM2.5 and AQI models, for example, a 0.7% versus a 0.6% increase for hypertension—provides further support for the robustness of the findings.

**Table 3:** Robustness test of changing independent variables

Variable	Hypertension	Diabetes	Dyslipidemia	Heart Disease	Stroke
AQI	0.006*** (0.001)	0.009*** (0.001)	0.012*** (0.001)	0.009*** (0.001)	0.007*** (0.002)
Control	Yes	Yes	Yes	Yes	Yes
Constant	−3.889*** (0.387)	−6.662*** (0.554)	−6.624*** (0.457)	−2.466*** (0.480)	−4.023*** (0.737)
Observation	15805	15806	15805	15805	15806

***represents *p* < 0.001

The second approach for robustness check is replacing models. Given that the five categories of CMDs are binary variables, the original logistic regression model was referenced, and probit regression was employed to analyze the impact of air pollution (PM2.5) on these diseases. The results, detailed in Table 4, demonstrate that the estimated coefficients retain statistical significance at the 1% level. For Diabetes coefficient, 0.006 means that OR is 1.006, which represents a 0.6% increase per AQI unit. It also provides further support for the robustness of the findings.

**Table 4:** Robustness test Replacement model Probit test

Variable	Hypertension	Diabetes	Dyslipidemia	Heart Disease	Stroke
PM2.5	0.004*** (0.001)	0.006*** (0.001)	0.010*** (0.001)	0.006*** (0.001)	0.005*** (0.001)
Control	Yes	Yes	Yes	Yes	Yes
Constant	−2.356*** (0.236)	−3.646*** (0.296)	−3.778*** (0.262)	−1.422*** (0.271)	−2.168*** (0.361)
Observation	15805	15806	15805	15805	15806

***represents *p* < 0.001

### Urban–rural heterogeneity analysis

To formally test whether the health impact of PM2.5 differs significantly between urban and rural residents, a pooled logistic regression model incorporating an interaction term between PM2.5 and a rural dummy variable was estimated, as specified in Eq. (3) as follows. The full results are presented in Table 5.

**Table 5:** Urban–Rural heterogeneity analysis

Variable	Hypertension	Diabetes	Dyslipidemia	Heart Disease	Stroke
PM2.5	0.004* (0.002)	0.008** (0.003)	0.012*** (0.002)	0.006** (0.002)	0.008* (0.010)
Rural	−0.256* (0.109)	−0.713*** (0.156)	−0.896*** (0.128)	−0.598*** (0.131)	−0.358# (0.202)
Pm2.5 × Rural	0.004 (0.002)	0.008* (0.003)	0.010*** (0.003)	0.007* (0.003)	0.005 (0.004)
Age	0.049*** (0.002)	0.025*** (0.003)	0.021*** (0.002)	0.049*** (0.002)	0.055*** (0.003)
Gender (ref: Man)					
Woman	0.034 (0.036)	0.248*** (0.050)	0.208*** (0.041)	0.578*** (0.044)	−0.078 (0.067)
Marital status (ref: no)					
Yes	0.026 (0.052)	0.172* (0.074)	0.123* (0.062)	0.176** (0.062)	−0.055 (0.088)
Education (ref: unschooled)					
Elementary school	−0.025 (0.044)	−0.041 (0.064)	0.081 (0.053)	0.184** (0.055)	0.088 (0.083)
Middle school and above	0.032 (0.045)	0.111# (0.063)	0.398*** (0.051)	0.399*** (0.055)	0.184* (0.084)
Social security (ref: no)					
Yes	0.199* (0.096)	0.442** (0.156)	0.461*** (0.126)	0.261* (0.123)	0.454* (0.204)
Household consumption	0.036* (0.018)	0.056* (0.026)	0.144*** (0.021)	0.065** (0.022)	0.084* (0.033)
GDP	−0.046* (0.019)	0.021 (0.027)	−0.020 (0.023)	−0.280*** (0.025)	−0.254*** (0.038)
Constant	−3.442*** (0.407)	−5.258*** (0.582)	−4.790*** (0.480)	−1.357** (0.503)	−3.322*** (0.775)
Observation	15805	15806	15805	15805	15806

*represents *p* < 0.05

**represents *p* < 0.01

***represents *p* < 0.001

3 $$\begin{aligned}&\mathrm{Cardiometabolic}\left(\mathrm{P}\left(\mathrm{Y}=1\right)\right)=\beta 0+\beta 1\mathrm{PM}2.5\\&+\beta 2\mathrm{Rural}+\beta 3\left(\mathrm{PM}2.5\times \mathrm{Rural}\right)+\delta \mathrm{X}\end{aligned}$$

The interaction term (PM2.5 × Rural) provides direct statistical evidence on the urban–rural differential in the health effects of PM2.5. For three of the five cardiometabolic diseases, namely diabetes (β = 0.008, *p* < 0.05), dyslipidemia (β = 0.010, *p* < 0.001), and heart disease (β = 0.007, *p* < 0.05)—the interaction coefficients are positive and statistically significant. This indicates that the adverse effect of PM2.5 exposure is significantly stronger for rural residents than for their urban counterparts concerning these specific conditions. For hypertension and stroke, the interaction terms are positive but not statistically significant at conventional levels (*p* > 0.05), suggesting that the effect of PM2.5 does not differ significantly between urban and rural residents for these diseases.

The calculation of total PM2.5 effects reveals meaningful urban–rural disparities. For diabetes, the PM2.5 effect is 0.008 for urban residents but increases to 0.016 for rural residents. For dyslipidemia, the effect rises from 0.012 for urban residents to 0.022 for rural residents. Similarly, for heart disease, the effect increases from 0.006 to 0.013.

Notably, the main effect of the rural dummy variable is negative and statistically significant for all five cardiometabolic diseases (hypertension: β = −0.256, *p* < 0.05; diabetes: β = −0.713, *p* < 0.001; dyslipidemia: β = −0.896, *p* < 0.001; heart disease: β = −0.598, *p* < 0.001; stroke: β = −0.358, *p* < 0.1). This indicates that, after controlling PM2.5 exposure and other covariates, rural residents exhibit significantly lower baseline risks of cardiometabolic diseases compared to urban residents. This paradoxical finding—characterized by a lower baseline risk but a greater vulnerability to PM2.5—merits careful interpretation.

For control variables, Age shows the expected positive association with all CMDs. The gender pattern varies by disease, with women showing higher risks for diabetes, dyslipidemia, and heart disease but not for hypertension or stroke. Higher household consumption is consistently associated with increased CMD risk across all five diseases. Higher education levels middle school and above) are associated with increased risks of dyslipidemia, heart disease, and stroke. Having social security coverage is associated with higher CMD odds across all diseases.

Several mechanisms may explain these observed patterns. First, the stronger PM2.5 effects in rural areas could stem from distinct pollution sources and compositions; rural PM2.5 often contains more toxic components originating from biomass burning and agricultural activities [5]. Second, rural residents may experience longer durations of outdoor exposure due to occupational patterns [6]. Third, lower healthcare access and health awareness in rural areas may reduce both preventive behaviors and the timeliness of treatment, thereby amplifying the impacts of pollution despite the lower baseline risks [4].

The contrast between lower baseline cardiometabolic disease risks and higher PM2.5 vulnerability among rural residents underscores a complex environmental health inequality. While rural populations may benefit from healthier lifestyles—such as greater physical activity and less consumption of processed foods—which reduce their baseline disease risk, they appear more susceptible to the additional risk imposed by air pollution. This heightened susceptibility may be attributable to compounded socioeconomic vulnerabilities and differing pollution characteristics.

### The moderating effect of medical resources

Theoretical analysis suggests that sufficient medical resources can reduce the risk of CMDs caused by air pollution, and per-capita physician availability in a region is an important indicator of the medical resources in the respondents’ living area. Therefore, we introduced an interaction term between PM2.5 and per-capita physician availability in the regression model in Eq. (2), to test whether the adequacy of regional medical resources plays a moderating role in the process of air pollution exacerbating the risk of CMDs.

Logistic regression analyses were conducted separately for the five categories of CMDs (see Table 6). The results indicate that the coefficients of interaction term related to hypertension, diabetes, dyslipidemia, heart disease, and stroke are statistically significantly negative at the 1% level, which means that for those areas with higher level of medical resources, the risk of CMDs caused by air pollution is reduced.

**Table 6:** Test of moderating effects

Variable	Hypertension	Diabetes	Dyslipidemia	Heart Disease	Stroke
PM2.5	0.025*** (0.005)	0.033*** (0.007)	0.068*** (0.006)	0.042*** (0.006)	0.035*** (0.010)
PPs	0.048*** (0.008)	0.043*** (0.012)	0.102*** (0.010)	0.095*** (0.010)	0.058*** (0.015)
Pm2.5 × PPs	−0.001*** (0.000)	−0.001** (0.000)	−0.002*** (0.000)	−0.001*** (0.000)	−0.001** (0.000)
Age	0.049*** (0.002)	0.026*** (0.003)	0.022*** (0.002)	0.050*** (0.002)	0.056*** (0.003)
Gender (ref: Man)					
Woman	0.030 (0.036)	0.267*** (0.050)	0.230*** (0.041)	0.578*** (0.044)	−0.076 (0.067)
Marital status (ref: no)					
Yes	0.018 (0.052)	0.152* (0.074)	0.094 (0.062)	0.158* (0.062)	−0.064 (0.088)
Education (ref: unschooled)					
Elementary school	−0.044 (0.044)	−0.006 (0.064)	0.108* (0.052)	0.148** (0.055)	0.076 (0.083)
Middle school and above	0.015 (0.044)	0.183** (0.062)	0.468*** (0.051)	0.370*** (0.054)	0.182* (0.083)
Social security (ref: no)					
Yes	0.190* (0.096)	0.434** (0.156)	0.439** (0.126)	0.250* (0.123)	0.448* (0.204)
Household consumption	0.041* (0.018)	0.082** (0.025)	0.178*** (0.021)	0.082*** (0.022)	0.095** (0.032)
GDP	−0.097*** (0.022)	0.023 (0.031)	−0.046# (0.025)	−0.401*** (0.028)	−0.307*** (0.042)
Constant	−3.971*** (0.451)	−7.120*** (0.660)	−7.916*** (0.551)	−2.172*** (0.566)	−4.242*** (0.867)
Observation	15,805	15,806	15,805	15,805	15,806

*represents *p* < 0.05

**represents *p* < 0.01

***represents *p* < 0.001

### Moderating effects between urban and rural areas

Although earlier analysis indicates that practicing physicians serves as a moderating variable in the relationship between air pollution (PM2.5) and CMDs, a more meaningful question is whether the supply of medical resources can become an important tool for alleviating health inequality between urban and rural areas.

To further investigate whether this moderating effect varies between urban and rural areas, logistic regression analyses were conducted separately for the five categories of CMDs in both settings (see Table 7).

**Table 7:** Moderating effects between urban and rural areas

Variable	Hypertension		Diabetes		Dyslipidemia		Heart Disease		Stroke	
	Urban	Rural	Urban	Rural	Urban	Rural	Urban	Rural	Urban	Rural
PM2.5	0.027** (0.008)	0.024*** (0.006)	0.028* (0.011)	0.036*** (0.010)	0.047*** (0.009)	0.084*** (0.009)	0.038** (0.010)	0.045*** (0.008)	0.020 (0.015)	0.045*** (0.013)
PPs	0.046*** (0.013)	0.048*** (0.010)	0.039* (0.017)	0.040* (0.017)	0.076*** (0.014)	0.119*** (0.014)	0.094*** (0.015)	0.093*** (0.013)	0.037 (0.023)	0.074*** (0.020)
PM2.5 × PPs	−0.001** (0.000)	−0.001*** (0.000)	−0.001# (0.000)	−0.001* (0.000)	−0.001*** (0.000)	−0.002*** (0.000)	−0.001** (0.000)	−0.001*** (0.000)	−0.0004 (0.000)	−0.001** (0.000)
Control	Yes	Yes	Yes	Yes	Yes	Yes	Yes	Yes	Yes	Yes
Constant	−4.312*** (0.747)	−3.406*** (0.600)	−5.172*** (1.006)	−6.768*** (0.938)	−5.072*** (0.828)	−8.228*** (0.792)	−0.420 (0.904)	−2.384** (0.777)	−2.696# (1.387)	−4.586*** (1.185)
Observation	6293	9512	6293	9513	6293	9512	6293	9512	6293	9513

*represents *p* < 0.05

**represents *p* < 0.01

***represents *p* < 0.001

The results in Table 7 demonstrate that, regardless of location, the moderating effect of practicing physicians on the coefficients for hypertension, dyslipidemia, and heart disease was statistically significant at the 1% level. However, for diabetes and stroke, the moderating effect was statistically significant at the 5% level in rural areas but not in urban areas. This heterogeneity in results may be associated with disease mechanisms, the saturation of medical effects, and urban–rural disparities. Overall, the findings suggest that in rural settings, the presence of practicing physicians significantly moderates the relationship between air pollution and CMDs among middle-aged and elderly individuals. In urban areas, this moderating role is limited to hypertension, dyslipidemia, and heart disease, with no significant effect observed for diabetes and stroke.

Consequently, it can be concluded that an increased per-capita physician availability enhances the availability of medical resources for both urban and rural residents, thereby reducing the likelihood of developing CMDs among middle-aged and elderly individuals. It suggests that sufficient medical resource supply can not only alleviate the risk of CMDs caused by air pollution, but also alleviate the current situation of urban–rural health inequality by more effectively benefiting rural residents.

## Discussion

This study builds upon existing research by utilizing 2018 cross-section datafrom the China Health and Retirement Longitudinal Study (CHARLS) and the annual average air pollution (PM2.5) index for prefecture-level cities across China. Logistic regression analysis was employed to examine the association between air pollution and the risk of CMDs among middle-aged and elderly individuals, to explore differences in disease risk between urban and rural residents, and to investigate the moderating effect of practicing physicians on the risk of CMDs due to air pollution. The key findings are as follows:

Air pollution (PM2.5) is significantly associated with CMDs in middle-aged and elderly individuals, and the conclusion remains valid following robustness testing. As best of our knowledge, this aligns with previous studies indicating a close association between air pollution and CMD risk in older demographics [12, 13, 28].

Compared to the health damage caused by air pollution, the urban–rural health inequality caused by air pollution is also worthy of attention. Urban–rural heterogeneity analysis revealed that rural residents experience greater health detriments than urban residents under equivalent levels of PM2.5 exposure, especially concerning dyslipidemia. This contradicts prior beliefs that urban residents are more severely affected by air pollution [3, 7, 8, 17, 24].

Contrary to the view that urban residents are more adversely affected, this study finds rural residents more susceptible to PM2.5-associated health risks. This disparity may be explained by several potential mechanisms. Rural residents are more likely to experience prolonged outdoor occupational exposure, such as agricultural work involving dust and pesticides [22]. They also tend to use less-protective transportation (e.g., motorcycles) and more polluting indoor fuels [14, 16, 19]. Moreover, lower incomes and weaker access to healthcare may reduce both protective behaviors and timely treatment among rural populations [2, 30]. These factors, though not directly tested here, suggest plausible pathways for the observed urban–rural health inequality.

The urban–rural health inequality stemming from air pollution may, however, be mitigated. Our mechanism analysis reveals that a higher density of practicing physicians in a region significantly attenuates the association between PM2.5 exposure and CMD risk among middle-aged and elderly residents. This suggests that enhancing medical resource supply—particularly the per-capita availability of physicians—could serve as a key policy lever to alleviate such disparities.

The moderating role of physicians likely operates through several pathways: improved access facilitates earlier disease management and slows progression; greater capacity supports community health education, raising awareness of pollution risks and self-protection [15]; and enhanced clinical resources allow for more precise diagnosis and treatment of pollution-related chronic conditions.

Besides, it is noteworthy that the moderating effect of the number of practicing physicians is not significant for diabetes and stroke risk in urban samples. This heterogeneity suggests that the protective role of medical resources may vary by disease type and urban context. Several explanations may account for this pattern.

First, regarding disease mechanisms, stroke—as an acute event—may involve vascular damage from long-term pollution exposure that is not easily reversed through routine outpatient care. In contrast, diabetes progression is strongly linked to urban environments characterized by obesogenic lifestyles, which may overshadow the moderating effect of clinical interventions.

Second, "medical effect saturation" may occur: in urban settings with already high physician density, the marginal preventive benefit of additional doctors may be limited, shifting the focus to factors such as quality of care, continuity, and patient adherence.

Finally, urban residents face a broader set of exposures—including noise, stress, and dietary patterns—which may interact synergistically with air pollution through multiple pathways, making it harder for clinical interventions alone to fully offset these combined risks.

This study possesses several marginal contributions. First, it expands empirical research on the impact of air pollution on health, for it leverages nationally representative CHARLS cross-section data matched with annual average PM2.5 levels at the prefecture-level city scale. Logistic regression was employed to control confounding factors such as individual characteristics, socioeconomic status, and regional indicators. Robustness tests (including variable substitution and model alterations) were conducted to ensure the reliability of the findings, providing a reference framework for data integration and methodological design in future studies. Second, the study contributes in empirical studies in urban–rural health inequality, for it demonstrates that rural residents suffer greater health impacts under the same air pollution exposure, explaining this heterogeneity through factors such as occupational nature, travel lifestyles, and economic/social support. This conclusion enriches the theoretical framework of “socially vulnerable groups” within environmental health research and highlights the spatial inequality in the health burden of air pollution. Third, it proposes possible policy instrument initiatives to alleviate urban–rural health inequality, by systematically confirming the moderating effect of practicing physicians on the health impacts of air pollution. It establishes that greater medical resource availability leads to a lower risk of CMDs attributable to air pollution. This contributes to the theoretical understanding of the “environment-health” relationship by incorporating medical resource accessibility into the explanatory mechanisms, offering a three-dimensional analytical framework of “environmental exposure, medical intervention, and health outcomes” for subsequent research.

In the meantime, the study has several notable limitations that merit acknowledgment. Using city-level annual average PM2.5 concentrations as a surrogate for individual exposure fails to capture within-city or interindividual exposure heterogeneity, and residential-based exposure assessment may lead to misclassification bias; meanwhile, disparities in activity patterns between urban and rural populations may introduce bias into cross-population comparisons of exposure effects. Furthermore, while annual average concentrations are suitable for investigating chronic health outcomes linked to long-term cumulative exposure, this aggregate metric does not reflect the potential adverse health impacts of short-term pollution peaks or seasonal air pollution variation. In addition, PM2.5 is a key component of the Air Quality Index (AQI), which renders the use of AQI for robustness checks subject to certain limitations. Additionally, physician density may be correlated with unobserved regional characteristics, and reverse causality may exist between physician density and the local number of chronic disease patients; such potential endogeneity issues may result in biased estimation results in our study. Future studies utilizing higher temporal resolution exposure data should explore whether pollution intensity or variability—beyond mean ambient levels—exert differential effects on the health of urban and rural populations.

Based on the empirical findings, three targeted policy recommendations are proposed. Firstly, our findings suggest implementing a rural-focused strategy for regionally differentiated pollution control. Given the stronger health effects of PM2.5 observed in rural areas, environmental policies should adopt spatially differentiated strategies. Recent research indicates that clean heating policies have significantly improved public health, particularly among middle-aged and older adults [10]. Instead of applying uniform standards, the following measures are recommended: Establishing air quality monitoring networks specifically for rural areas, with a focus on emissions from biomass burning and agricultural activities; Implementing targeted subsidy programs to promote the adoption of clean heating and cooking technologies in rural households; Incorporating the urban–rural differential in health impacts into the assessment framework for pollution control policies.

Secondly, our findings advocate for implementing a physician-resource-centered medical investment mechanism in rural areas. The moderating effect analysis provides direct evidence that increased physician density mitigates the health risks associated with pollution. Consequently, it is recommended that: Physician recruitment and retention in rural regions be prioritized over general healthcare infrastructure investments; Specialized training programs for "environmental health physicians," focused on the management of pollution-related diseases, be developed; Financial incentives be established for physicians who practice in high-pollution rural areas.

Thirdly, our findings recommend establishing an integrated collaborative governance model for environment and health. The interaction between pollution exposure and medical resource availability underscores the need for coordinated governance. This should include joint planning between environmental and health departments in resource allocation; health impact assessments that explicitly account for local medical resource availability; and targeted cardiometabolic disease screening programs in high-pollution rural communities with limited healthcare access.

## Data Availability

The datasets analysed during this study are all publicly available from the CHARLS sources: https://charls.pku.edu.cn/en/.

## References

[1] Bradley AC, Croes BE, Harkins C, McDonald BC, de Gouw JA. Air Pollution Inequality in the Denver Metroplex and its Relationship to Historical Redlining. Environ Sci Technol. 2024;58(9):4226-36. 10.1021/acs.est.3c03230. 10.1021/acs.est.3c03230 PMC10919081 38380822

[2] Brochu PJ, Yanosky JD, Paciorek CJ, Schwartz J, Chen JT, Herrick RF, et al. Particulate air pollution and socioeconomic position in rural and urban areas of the Northeastern United States. Am J Public Health. 2011;101(1(Suppl 1)):S224–30. 10.2105/AJPH.2011.300232. 21836114 10.2105/AJPH.2011.300232 PMC3222475

[3] Brønnum-Hansen H, Bender AM, Andersen ZJ, Sørensen J, Bønløkke JH, Boshuizen H, et al. Assessment of impact of traffic-related air pollution on morbidity and mortality in Copenhagen Municipality and the health gain of reduced exposure. Environ Int. 2018;121(1):973–80. 10.1016/j.envint.2018.09.050. 30408890 10.1016/j.envint.2018.09.050

[4] Chai J, Chen P, Feng R, et al. Life events and chronic physical conditions among left-behind farmers in rural China a cross-sectional study. BMC Public Health. 2015;15:594. 10.1186/s12889-015-1877-0. 26130045 10.1186/s12889-015-1877-0 PMC4487061

[5] Chan KH, Xia X, Ho KF, et al. Regional and seasonal variations in household and personal exposures to air pollution in one urban and two rural Chinese communities: a pilot study to collect time-resolved data using static and wearable devices. Environ Int. 2021;146:106217. 10.1016/j.envint.2020.106217. 33129001 10.1016/j.envint.2020.106217 PMC7786640

[6] Du W, Li XY, Chen YC, Shen GF. Household air pollution and personal exposure to air pollutants in rural China: a review. Environ Pollut. 2018;237:625–38. 10.1016/j.envpol.2018.02.054. 29525629 10.1016/j.envpol.2018.02.054

[7] Huang B, Xiao T, Grekousis G, Zhao H, He J, Dong G, et al. Greenness-air pollution-physical activity-hypertension association among middle-aged and older adults: evidence from urban and rural China. Environ Res. 2021;195:110836. 10.1016/j.envres.2021.110836. 33549617 10.1016/j.envres.2021.110836

[8] Ing C, Beattie C, Longhurst J. Progress with implementing local air-quality management in rural areas of England. J Environ Manage. 2001;61(2):137–47. 10.1006/jema.2000.0391. 11381771 10.1006/jema.2000.0391

[9] Li C, Qi J, Yin P, Yu X, Sun H, Zhou M, et al. The burden of type 2 diabetes attributable to air pollution across China and its provinces, 1990-2021: an analysis for the Global Burden of Disease Study 2021. Lancet Regional Health - Western Pacific. 2024;53:101246. 10.1016/j.lanwpc.2024.101246. 39655197 10.1016/j.lanwpc.2024.101246 PMC11626817

[10] Liao L, Kong S, Du M. The effect of clean heating policy on individual health: evidence from China. China Econ Rev. 2025;89:102309.10.1016/j.chieco.2024.102309.

[11] Liu Haimeng. Annual Air Quality Index (AQI) of 367 Chinese Cities (2014–2024)[DS/OL]. V1. Science Data Bank, 2025. https://cstr.cn/31253.11.sciencedb.20642.CSTR:31253.11.sciencedb.20642.

[12] Liu M, Tang W, Zhang Y, Wang Y, Baima Kangzhuo, Li Y, Liu X, Xu S, Ao L, Wang Q, Wei J, Chen G, Li S, Guo Y, Yang S, Han D, Zhao X; China Muti-Ethnic Cohort (CMEC) collaborative group. Urban-rural differences in the association between long-term exposure to ambient air pollution and obesity in China. Environ Res. 2021;201:111597. 10.1016/j.envres.2021.111597. Epub 2021 Jun 29. PMID: 34214564. 10.1016/j.envres.2021.111597 34214564

[13] Luo H, Zhang Q, Yu K, Meng X, Kan H, Chen R. Long-term exposure to ambient air pollution is a risk factor for trajectory of cardiometabolic multimorbidity: a prospective study in the UK Biobank. EBioMedicine. 2022;84:104282. 10.1016/j.ebiom.2022.104282. 36174399 10.1016/j.ebiom.2022.104282 PMC9520206

[14] Mestl HE, Aunan K, Seip HM, Wang S, Zhao Y, Zhang D. Urban and rural exposure to indoor air pollution from domestic biomass and coal burning across China. Sci Total Environ. 2007;377(1):12–26. 10.1016/j.scitotenv.2007.01.087. 17343898 10.1016/j.scitotenv.2007.01.087

[15] Mirabelli MC, Damon SA, Beavers SF, Sircar KD. Patient-provider discussions about strategies to limit air pollution exposures. Am J Prev Med. 2018;55(2):e49–52. 10.1016/j.amepre.2018.03.018. 29903566 10.1016/j.amepre.2018.03.018 PMC6075706

[16] Mohajeri N, Hsu SC, Milner J, Taylor J, Kiesewetter G, Gudmundsson A, et al. Urban-rural disparity in global estimation of PM2·5 household air pollution and its attributable health burden. Lancet Planetary Health. 2023;7(8):e660–72. 10.1016/S2542-5196(23)00133-X. 37558347 10.1016/S2542-5196(23)00133-X PMC10958988

[17] Niu L, Zhang Z, Liang Y, van Vliet J. Spatiotemporal patterns and drivers of the urban air pollution island effect for 2273 cities in China. Environ Int. 2024;184:108455. 10.1016/j.envint.2024.108455. 38277996 10.1016/j.envint.2024.108455

[18] Rajagopalan S, Brook RD, Salerno PRVO, Bourges-Sevenier B, Landrigan P, Nieuwenhuijsen MJ, et al. Air pollution exposure and cardiometabolic risk. Lancet Diabetes Endocrinol. 2024;12(3):196–208. 10.1016/S2213-8587(23)00361-3. 38310921 10.1016/S2213-8587(23)00361-3 PMC11264310

[19] Samoli E, Atkinson RW, Analitis A, Fuller GW, Beddows D, Green DC, et al. Differential health effects of short-term exposure to source-specific particles in London, U.K. Environ Int. 2016;97:246–53. 10.1016/j.envint.2016.09.017. 27692926 10.1016/j.envint.2016.09.017

[20] Sarkar C, Lai KY. Urban built environments: interventions for reducing cardiometabolic risks. Nat Rev Endocrinol. 2023;19(6):315–6. 10.1038/s41574-023-00827-2. 36997806 10.1038/s41574-023-00827-2

[21] Stieb DM, Smith-Doiron M, Quick M, Christidis T, Xi G, Miles RM, et al. Inequality in the distribution of air pollution attributable mortality within Canadian cities. Geohealth. 2023;7(9):e2023GH000816. 10.1029/2023GH000816. 37654974 10.1029/2023GH000816 PMC10465848

[22] Sun D, Liu C, Ding Y, Yu C, Guo Y, Sun D, et al. Long-term exposure to ambient PM2·5, active commuting, and farming activity and cardiovascular disease risk in adults in China: a prospective cohort study. Lancet Planet Health. 2023;7(4):e304–12. 10.1016/S2542-5196(23)00047-5. 37019571 10.1016/S2542-5196(23)00047-5 PMC10104773

[23] Waard AM, Hollander M, Korevaar JC, Nielen MMJ, Carlsson AC, Lionis C, Seifert B, Thilsing T, Wit NJ, Schellevis FG, the SPIMEU Project Group. Selective prevention of cardiometabolic diseases: activities and attitudes of general practitioners across Europe. European Journal of Public Health. 2019;29(1):88–93. 10.1093/eurpub/cky112. 10.1093/eurpub/cky112 PMC6345147 30016426

[24] Wang J, Lin J, Liu Y, Wu F, Ni R, Chen L, Ren F, Du M, Li Z, Zhang H, Liu Z. Direct and indirect consumption activities drive distinct urban-rural inequalities in air pollution-related mortality in China. Sci Bull (Beijing). 2024;69(4):544-53. 10.1016/j.scib.2023.12.023. 10.1016/j.scib.2023.12.023 38158290

[25] Wei J, Li Z, Lyapustin A, Sun L, Peng Y, Xue W, Su T, Cribb M. Reconstructing 1-km-resolution high-quality PM2.5 data records from 2000 to 2018 in China: spatiotemporal variations and policy implications. Remote Sensing of Environment. 2021;252:112136. 10.1016/j.rse.2020.112136.

[26] World Health Organization (2020) Air pollution. World Health Organization. http://who.int/health-topics/air-pollution World

[27] Yu Z, Su L, Ni P, He J, Su Y, Cui J, et al. Impact of air pollution and behavioral factors on cognitive decline among middle-aged and elderly populations in China: a retrospective cohort study based on CHARLS. BMC Public Health. 2025;25(1):2198. 10.1186/s12889-025-23211-3. 40604741 10.1186/s12889-025-23211-3 PMC12219787

[28] Zhao X, Sun Z, Ruan Y, Yan J, Mukherjee B, Yang F, et al. Personal black carbon exposure influences ambulatory blood pressure: air pollution and cardiometabolic disease (AIRCMD-China) study. Hypertension. 2014;63(4):871–7. 10.1161/HYPERTENSIONAHA.113.02588. 24420543 10.1161/HYPERTENSIONAHA.113.02588 PMC4445364

[29] Zhao Q, Feng Q, Seow WJ. Impact of air pollution on depressive symptoms and the modifying role of physical activity: evidence from the CHARLS study. J Hazard Mater. 2025;482:136507. 10.1016/j.jhazmat.2024.136507. 39579693 10.1016/j.jhazmat.2024.136507

[30] Zhu J, Lu C. Air quality, pollution perception, and residents’ health: evidence from China. Toxics. 2023;11(7):591. 10.3390/toxics11070591. 37505557 10.3390/toxics11070591 PMC10383338

